# The role of wearables in spinal posture analysis: a systematic review

**DOI:** 10.1186/s12891-019-2430-6

**Published:** 2019-02-08

**Authors:** Lauren Simpson, Monish M. Maharaj, Ralph J Mobbs

**Affiliations:** 1 NeuroSpine Surgery Research Group (NSURG), Sydney, Australia; 2 Faculty of Medicine, University of New South Wales, Sydney, Australia; 3 Department of Neurosurgery, Prince of Wales Hospital, Sydney, Australia; 4 Prince of Wales Hospital, Randwick, NSW Australia

**Keywords:** Wearable technology, Postural assessment, Patient outcomes, Spine posture

## Abstract

**Background:**

Wearables consist of numerous technologies that are worn on the body and measure parameters such as step count, distance travelled, heart rate and sleep quantity. Recently, various wearable systems have been designed capable of detecting spinal posture and providing live biofeedback when poor posture is sustained. It is hypothesised that long-term use of these wearables may improve spinal posture.

**Research questions:**

To (1) examine the capabilities of current devices assessing spine posture, (2) to identify studies implementing such devices in the clinical setting and (3) comment on the clinical practicality of integration of such devices into routine care where appropriate.

**Methods:**

A comprehensive systematic review was conducted in adherence to the Preferred Reporting Items for Systematic Reviews and Meta-Analyses Guidelines (PRISMA) across the following databases: PubMed; MEDLINE; EMBASE; Cochrane; and Scopus. Articles related to wearables systems able to measure spinal posture were selected amongst all published studies dated from 1980 onwards. Extracted data was collected as per a predetermined checklist including device types, study objectives, findings and limitations.

**Results:**

A total of 37 articles were extensively reviewed and analysed in the final review. The proposed wearables most commonly used Inertial Measurement Units (IMUs) as the underlying technology. Wearables measuring spinal posture have been proposed to be used in the following settings: post-operative rehabilitation; treatment of musculoskeletal disorders; diagnosis of pathological spinal posture; monitoring of progression of Parkinson’s Disease; detection of falls; workplace occupational health and safety; comparison of interventions.

**Conclusions:**

This is the first and only study to specifically review wearable devices that monitor spinal posture. Our findings suggest that currently available devices are capable of assessing spinal posture with good accuracy in the clinical setting. However, further validation regarding the long-term use of these technologies and improvements regarding practicality is required for commercialisation.

## Introduction

Wearables are defined as electronic technologies or computers that can be worn on the body. Since the beginning of the twenty-first century such devices have reduced in size and costs while improving in observation capabilities related to multiple health-related parameters, triggering a shift towards commercialisation and incorporation into everyday activities [1]. In 2016, there were 61-million wearables monitoring physical activity online, forecasted to reach 187 million by 2020 [2]. The increased uptake of these devices has contributed to the self-monitoring movement of health. Most current wearable systems are accompanied by smartphone applications that permit users to engage and promote self-awareness of their behaviours as a means of motivating improvement and taking personal responsibility for their health [3]. Outside of physical activity, newer systems include observation of heart rate, blood pressure, posture and sleep [4]. This present review aims to focus on wearables analysing spinal posture and its health-related implications.

There is a need for real-time postural monitoring and correction as sustained poor spinal posture is associated with the development and worsening of many musculoskeletal disorders [4, 5]. Poor spinal posture, as defined by Hansraj et al. [6], relates to the relative position whereby the head and upper trunk is in a forwards-flexed position. Neutral posture is considered when the head and upper trunk is at zero degrees to the rest of the spine, with subsequent increase in angle correlating with poorer posture and its associated complications. [6]. These complications are hypothesized to included improper alignment of vertebrae, intervertebral disc damage/degeneration and nerve root impingement. Clinically, this may be linked to symptoms including neck and back pain, radiculopathy and sensorimotor deficit. In severe symptomatic case this may warrant surgical intervention [7]. Ultimately, poor posture can lead to a significant economic burden through increased healthcare costs and lost productivity within the workforce [4]. While it is difficult to estimate the total cost of poor posture alone, the predicted burden of back pain in the United States alone as estimated by Shekelle et al. [8] is an annual net cost of $60 billion.

Wearables monitoring posture have the potential to prevent the aforementioned consequences through real-time biofeedback encouraging the correction of sustained poor posture. It is hypothesised that with long-term use these systems may instil correct postural habits and yield a decrease in the incidence of posture-related musculoskeletal disorders. In a systematic review by Wang et al. [9] focused on sensor technology feedback systems of the upper limb (*n* = 42 studies) overall validation studies for multiple systems have been favourable, although clinical integration of these systems has not yet materialised on a large scale. In a separate review by McCallugh et al. [10] in the ageing and hospitalised population, a subset analyses on spinal posture across 6 devices had suggested high accuracy readings, although the use of combined feedback systems to target change and rehabilitation was generally lacking and remains an ongoing focus. The study was limited to devices detecting changes in sitting and standing posture, with further analyses outside the scope of the published review.

Conventionally, spinal posture is assessed in a clinical setting during routine patient examinations, physiotherapy sessions or formalised laboratory based evaluation. Such traditional methods have been criticised as costly and impractical with a key inability to measure day-to-day posture and provide timely feedback. The gold standard for analysing spinal posture remains radiographical assessment, however the associated cost and irradiation limits frequent repeated use [5]. Other lab-based modalities include: goniometers, photogrammetric systems and optoelectric systems may be utilised but have failed to integrate into routine medical practice.

This is the first study to review the use of wearables in spinal posture monitoring with aims to (1) examine the capabilities of current devices, (2) identify studies implementing such devices in a clinical setting and (3) comment on the clinical practicality of integration of such devices into routine clinical care. Our findings aim to synthesise the volume of data published across multiple scientific domains (engineering, computer science, rehabilitation medicine etc) and streamline a method of customised device design, in order to optimise the clinical use of postural sensors and ultimately use the data they can provide to facilitate clinical decision making such as the need for surgical intervention.

## Methods

### Literature search

The Preferred Reporting Items for Systematic Reviews and Meta-Analyses Guidelines (PRISMA) were adhered to for this systematic review across the following five databases: PubMed; MEDLINE; EMBASE; Cochrane; and Scopus [11]. The final choice of key search terms was derived from pre-established headings on the OVID Medline database after a generic screening using a list of relevant key terms. Key search terms included: ‘Monitoring, Ambulatory’ OR ‘Wearable Electronic Devices’ OR ‘Wearables’ AND ‘Posture’. Relevant MeSH (Medical Subject Heading) terms, spelling variations and synonyms were included and modified as appropriate for each database. Studies addressing both wearable systems and posture were selected. The PRISMA flow chart is illustrated in figure.

### Study selection

Inclusion and exclusion criteria are documented below. Duplicate studies were removed, with journal papers chosen over conference papers in the setting of data duplication. In cases where multiple studies included the same dataset, the most recent update was included. A primary screening was conducted by an independent reviewer (LS) who read the full texts of selected articles based on the inclusion and exclusion criteria; (4) A secondary screening was conducted by an independent reviewer (MM) who read the full texts of selected articles based on the inclusion and exclusion criteria. In the setting of discrepancy in study inclusion the senior author (RM) authorised the final decision.

Inclusion criteria:

1. Articles involving wearable technology/ies
2. The wearable technology/ies are able to monitor posture in the sagittal and/or coronal planes
3. The wearable technology/ies are able to monitor posture of the spine
4. Articles written in English
5. Time of publication between 1980- April 2018.

Exclusion criteria:

1. Wearable technology/ies only capable of identifying activity or discriminating between body position (e.g. sitting, standing lying)
2. Wearable technology/ies only capable of monitoring posture of body parts other than the spine
3. Wearable technology/ies classed as robotic or exoskeletons
4. Systematic reviews
5. Books

### Data collection

Following the selection of articles, data was collated by two researchers (LS and MM). Data was collected as per a predetermined checklist including: type of wearable technology/ies; sensor location/s; presence of feedback system; aims of the study; and outcomes of the study. All included articles were appraised for bias using the Newcastle-Ottawa Scale of Quality Assessment.

## Results

**Fig. 1:**
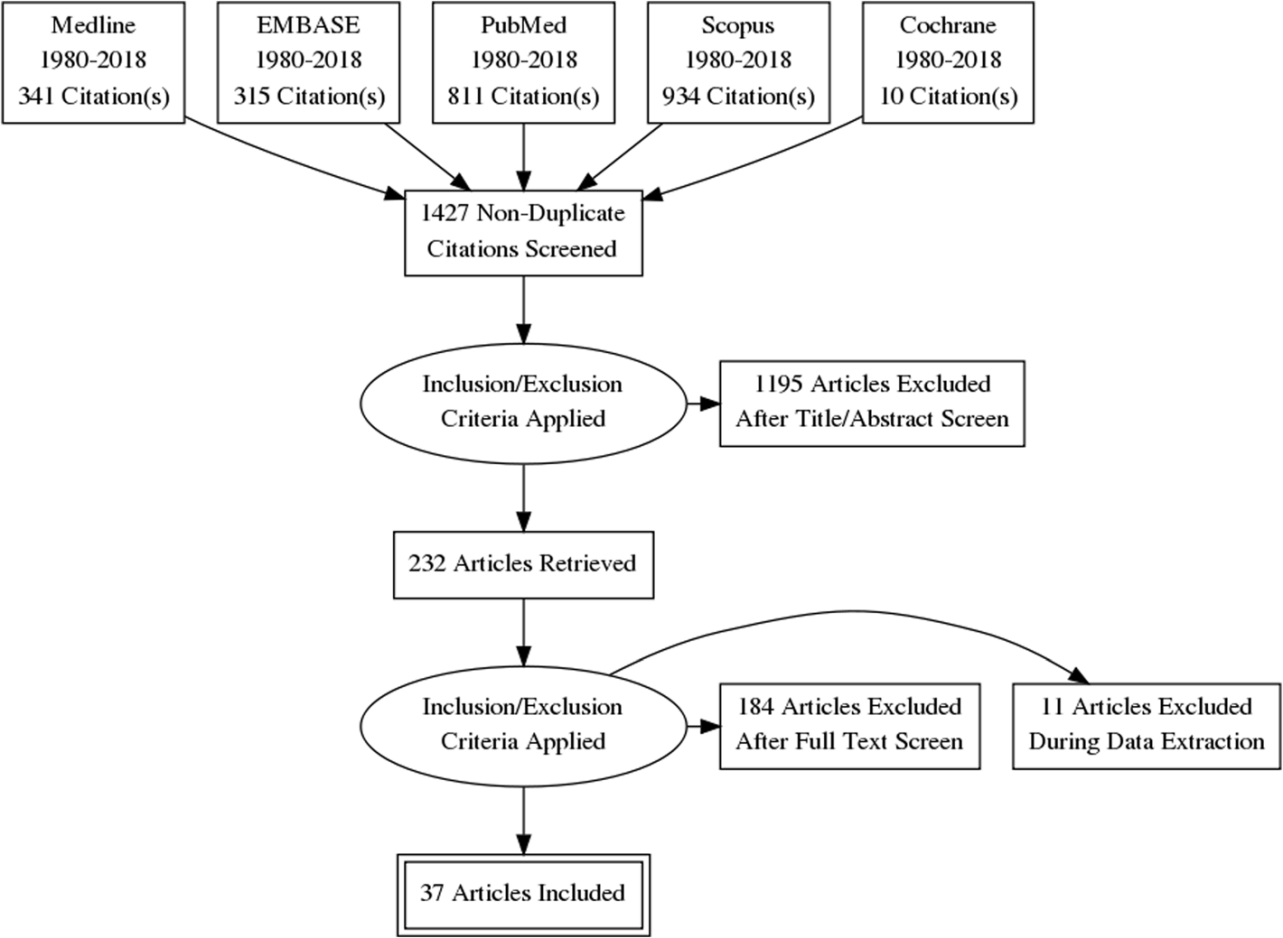
PRISMA Flowchart outlining literature search and study selection

From the 1427 non-duplicate articles found using the search strategy, 37 articles were selected for inclusion (Fig. 1). Summarised findings from selected articles are included in Table 1.

**Table 1:** Summary of results from reviewed articles and identified limitations

Reference	Wearable technology/ies	Sensor location/s and error rate (ER)	Feedback system	Aims of study	Conclusions of study	Key limitations from bias assessment and conclusions
Nath et al., 2017 [36]	Smartphone IMU	1: upper arm ER: 0–6% 2: waist ER: 0–1%	No real-time feedback	Validation of built-in smartphone IMUs to measure workers’ postures and identify risks	Calculated postures close to observation-based methods; reliable method for identifying postural risks and trunk flexion	Comparability limitation: Only tested in the context of 16 screw driving scenarios in one worker. More variable error rate in arbitrary position readings.
O’Sullivan et al., 2012 [17]	BodyGuard: strain gauge	From spinous process of L3 to S2 calibrated to individual based on %ROM - correlation to digital fluoroscopy: sitting vs standing *r*^2^ = 0.94 vs 0.88	Real-time biofeedback (auditory or visual)	Validation of BodyGuard for analysis of vertebral motion in the sagittal plane (*n* = 12)	Slight and consistent underestimate of lumbopelvic flexion; validated method for use in laboratory and clinical settings.	Outcome limitations: Further validation required for use in individuals with low back pain
Bhattacharya et al., 1999 [18]	Ergonomic dosimeter	Trunk and upper dominant arm (housed in coveralls) - Output stratified into risk categories based on ROM magnitude	No real-time feedback	Validation of system to measure postural angles of torso and upper arm in sagittal plane (*n* = 2)	Reliable system for the continuous monitoring of postural data in carpenters on construction sites	Selection bias: Small cohort not representative of general population No data on correlation to video data during scenarios
Plamondon et al., 2007 [15]	Hybrid system: two IMUs linked by potentiometer	IMUs: 1: S1 (pelvic analysis) 2: T1 spinous process (thoracic flexion/lateral flexion and torsion) - Error in degrees: 2.7, 1.9 and 5.2 respectively	No real-time feedback	Validation of hybrid system for 3D measurement of trunk posture; analysis of utility of potentiometer to increase validity (*n* = 6)	Root mean square error less than 3 degrees for forward- and lateral-flexion; potentiometer required when magnetometer signals corrupted	Comparability and outcome limitation: Error increased in long-duration dynamic tests (30 min) vs. short-duration (30 s) particularly without magnometer
Faber et al., 2009 [37]	MTx IMU System	1: sacrum 2: T9 3: movable (between 1 and 2) Peak error rate ¬5 degrees	No real-time feedback	Determination of the possibility and optimal location of a single sensor for trunk inclination measurement (*n* = 10)	Optimal inertial sensor location for trunk inclination measurement 25% of the distance from the midpoint between the PSISs to C7 and was hence different to each subject.	Comparability bias: tested with straight legs; flexion of knees may impact trunk inclination when lifting an object, hence the optimal location may change
Gleadhill et al., 2016 [38]	SABEL Sense IMU	1: C7 2: T12 3: S1	No real-time feedback	Validation of inertial sensors for measurement of resistance exercise movement patterns (deadlift). 11 subjects provided 227 time points to analyse.	Timing validation results demonstrated a Pearson’s correlation of 0.9997 and supportive validity measures; validated for use in resistance exercise	Comparability bias: Only tested in the context of a conventional deadlift with ROM not specified
Yan et al., 2017 [24]	YEI 3-Space IMU Sensor	1: back 2: safety helmet	Real-time auditory alarm	Validation of a personal protective equipment involving IMUs for insecure motion warning	Successful validation of the proposed technology for real-time insecure motion warning	No comparison to analyse accuracy and no formal published output data provided.
Fathi et al., 2017 [34]	Shimmer IMU	1: cervical spine 2: thoracic spine 3: lower lumbar spine Reported accuracy rate of 100% across pre-defined stages of ankylosing spondylitis	Reported real-time feedback but mechanism of the same not detailed	Proposal of wearable system able to detect spinal displacement and provide real-time warnings	System classification performance validated in differentiating between two incorrect postures (hunch back, slouch back)	Selection bias: Only evaluated in four subjects, no information regarding their health or tasks performed was provided
Abyarjoo et al., 2015 [14]	PostureMonitor: YEI 3-Space IMU Sensor	Attached to upper back of the user’s garment	Real-time auditory alarm	Verification of the PostureMonitor for the detection of poor posture and development of good postural habits	PostureMonitor reported sensitive as to detect and warn of poor posture.	Outcome limitation: further testing required for validation, long-term testing required to assess the impact on the development of good postural habits
Cajamarca et al., 2017 [5]	StraightenUp: LilyPad Accelerometer ADXL335	Sensors attached to a brace: 1: upper trunk 2: central trunk 3: lower trunk Precision rate across different pre-defined positions ranged from 99 to 100% (*n* = 9000)	No real-time feedback	Verification of StraightenUp for measurement of spinal posture and assessment of user experience (*n* = 30, 9000 encounters)	Preliminary verification of postural classification; reported to be comfortable but difficult to apply; user preference for vibrotactile or smartphone notification for poor posture alerts	Outcome limitation: Further testing required for validation; device requires adaptation to become more user friendly Not tests in real life setting
Valdivia et al., 2017 [39]	IMU MPU-9250 sensor	Sensor strapped to elastic band worn at the waist	Real-time feedback via exergame	Comparison of IMU sensor with Microsoft Kinect V2 for the use in a proposed exergame aimed at improving spinal posture	IMU more accurately but less reliably measures range of motion of the spine in comparison with the Microsoft Kinect V2; IMU exergame less engaging	Selection bias: Comparison of IMU and Microsoft Kinect between different subjects in an already low sample size
Wang et al., 2016 [26]	Zishi: 9-axis Adafruit IMU sensor	Two sensors within a vest: 1: T1 2: T5 Root mean square error range 2–5 degrees	Real-time visual and auditory feedback via Android app	Development, validation and incorporation of the Zishi in postural analysis and correction	Fifth iteration for the Zishi vest provided highly mobile smart textile for postural analysis	Outcome limitation: Further validation studies recommended; expansion to measure aspects of spinal posture (e.g. lumbar region) useful for better analysis of posture
Tanaka et al., 1994 [12]	Electromagnetic inclinometer LP06F1F1AA Murata	1: chest 2: thigh 3: leg	No real-time feedback	Proposal of wearable system for long-term measurement of human posture	Device able to record postural changes with an angular resolution of 12 degrees. No accuracy or error data provided.	Outcome limitation: Angular resolution inadequate for precise measurement; limited to sagittal plane
Wong et al., 2008 [33]	IMU: one tri-axial accelerometer and three uni-axial gyroscopes	Sensors strapped with elastic: 1: T1/T2 2: T12 3: S1 Error rate in postural assessment: < 3 degrees in sagittal and coronal planes, ICC > 0.829	Real-time auditory alarm	Proposal of posture monitoring system able to estimate spinal curvature changes in sagittal and coronal planes and provide postural analysis (*n* = 9)	Preliminary verification indicated high correlation with motion analysis system; verified for remote monitoring of trunk posture during daily activities	Outcome limitation: Lack of magnetometer did not allow for estimation of trunk rotation in transverse plane
Xu et al., 2017 [40]	9-axis IMU: MPU-9150 InvenSense	Eight IMUs placed symmetrically on left and right sides of torso at L4/L5	Real-time vibrotactile feedback	Proof-of-concept of wearable system for real-time postural balance and gait retraining using vibrotactile feedback (*n* = 4 and 6 in 2 studies)	Device able to monitor trunk tilt and provide meaningful vibrotactile feedback	Outcome limitation: Further testing required for validation as the current study was a proof-of-concept; battery life of IMUs only 1.5 h Raw error rates not provided.
Bazzarelli et al., 2003 [28]	Hybrid system: electromagnetic technology and Analog Devices ADXL202 biaxial accelerometer	1: left scapula 2: right scapula RMS error 1%	Real-time vibrotactile feedback	Proposal of hybrid system to replace braces in the correction of adolescent idiopathic scoliosis (*n* = 6)	Preliminary verification of hybrid system for monitoring progress and correction via biofeedback in adolescent idiopathic scoliosis with good sensitivity.	Outcome limitation: Further testing required for validation. No current data in real user.
Dunne et al., 2008 [41]	Plastic fibre-optic goniometer	Markers placed on C7, T4, T7, T10, T12, L2, L4 + spines of left and right scapulae	No real-time feedback	Validation of plastic optical fibre sensor for monitoring seated spinal posture, as compared to visual analysis (*n* = 9)	Significant accuracy error ranging across 14.5% of the magnitude of the average range of motion of subjects	Outcome limitation: Further testing required for validation in clinical contexts No error data provided.
Motoi et al., 2006 [42]	IMU: accelerometer and gyroscope	1: chest, housed in shirt pocket 2: lower thigh 3: upper calf	No real-time feedback	Proposal of wearable system for monitoring gait speed and angle changes of trunk, thigh and calf in sagittal plane (*n* = 3)	Preliminary verification of use of the wearable system for dynamic posture monitoring in sagittal plane	Comparison and outcome limitations: Poor wearability with sensors linked by a wire No error rate comparison
Gopalai et al., 2012 [43]	MicroStrain’s wireless IMU	1: Attached to trunk via waist band 2: wobble board	Real-time vibrotactile feedback	Evaluation of real-time vibrotactile feedback for the warning of poor postural control (*n* = 24)	Preliminary verification of detection of poor postural control; improved postural control with vibrotactile feedback	Comparability limitation: Less related to spinal posture monitoring and more focused on postural stability using feedback system
Wu et al., 2014 [30]	Accelerometer	Vest containing: 1: below neck 2: chest 3: centre of mass 4: left hip 5: right hip Angle errors within 0.5 degrees	No real-time feedback	Proposal of using multiple single-axis accelerometers to obtain titling angles (*n* = 20)	Wearable system and time-less algorithm proposed verified for real-life applications	Outcome Limitation: Further testing required for validation in the suggested context (Parkinson’s disease) and other clinical contexts
Sardini et al., 2015 [19]	Inductive sensor	Shirt with an inductive sensor sewn to the back and front Correlation coefficients range from 0.95–0.98 to optical system.	Real-time vibrotactile feedback	Validation of wearable system for monitoring seated posture at home through comparison with optical measuring system (*n* = 4)	Validated for the use of monitoring seating posture in a variety of functional activities within the home	Outcome limitation: Only measures spinal posture in sagittal plane; further testing in a greater variety of contexts required for wider validation
Tsuchiya et al., 2015 [20]	Flex sensor + accelerometer	Accelerometers (2) placed at upper lumbar spine + sacrum, flex sensors (3) placed between	No real-time feedback	Proposal of wearable system to measure the shape of lumbar skin to identify lumbosacral alignment changes in 3 positions xray (*n* = 4)	Lumbosacral alignment and lumbar load accurately estimated using wearable system	Comparability limitation: Less related to spinal posture monitoring and more focused on lumbosacral dimension estimation
Miyajima et al., 2015 [44]	Six-axis IMU: accelerometer and gyroscope across knee, hip and spine.	1: lumbar spine 2: thigh 3: calf Mean angle error < 3.5 degrees across sensors	No real-time feedback	Verification of wearable system for monitoring lumbar torque through comparison with optical capture system (*n* = 1)	Estimation error of lumbar joint torque < 11 Nm based on inclination angle data; preliminarily verified.	Comparability limitation: Assumption that all angles were at 0 degrees when subjects were standing straight. More subjects needed.
Petropoulos et al., 2017 [6]	SPoMo: six-axis IMU (accelerometer and gyroscope)	1: upper back 2: lower back Mean square error range: 0.001–0.05	Real-time vibrotactile feedback	Proposal of SPoMo for the real-time automatic monitoring of spinal posture in sitting	Average mean square error suggests SPoMo is a reliable tool for monitoring sitting spinal posture	Comparability limitation: Accumulated error due to gyroscope drift, requires well refined calibration and filtering of data for long-term use
Lou et al., 2012 [29]	Smart garment: IMU (three-axis accelerometer and two-axis gyroscope)	1: upper back 2: lower back Error in static measurements of 2 degrees	Real-time vibrotactile feedback	Verification of smart garment for posture monitoring during daily activities; analysis of efficacy of vibrotactile feedback compared to video (*n* = 4)	Measurement accuracy within 5 degrees over 90% of the time during daily activities	Outcome limitation: No indication of whether long-term use with vibrotactile feedback can lead to long-term postural change Data only on single plane kyphosis measured.
Bell et al., 2007 [21]	Fibre-optic goniometer	L5/S1 No data on error rate or accuracy.	No real-time feedback	Proposal of wearable system using fibre-optic goniometers to identify activities and associated lumbar postures (*n* = 5)	System reported as comfortable and unobtrusive; motion profiles accurately identified work-related activities and quantify lumbar postures	Outcome limitation: Postural identification is not currently automated in the proposed system, preventing real-time feedback restricting usability. No comparison data for accuracy.
Ribeiro et al., 2016 [45]	Spineangel: triaxial accelerometer	Attached to belt	Real-time auditory alarm	Investigation of the extent to which the Spineangel can reduce exposure to poor posture associated with low back pain	Within-day measurement error of 5 degrees and between-day measurement error of 8 degrees	Outcome limitation: Study published was a protocol for the ELF cluster randomised controlled trial, results not yet published
Harms et al., 2009 [16]	SMASH accelerometers	Fixed on shirt: 1: C7 2: T10 3: L5 4: scapula 5: shoulder Absolute sensor error less than 5 degrees in 84% of cases.	No real-time feedback	Validation of system involving accelerometers fixed to shirt to measure trunk inclination in children, as compared with vision-based system (*n* = 21 subjects across 6 positions)	Single scapula sensor most valuable in assessing Posture based on the least error derived	Comparability limitation: The shirt to which the sensors were affixed was loose fitting, thus allowing sensor movement and subsequent error particularly in setting of head movement and significant trunk flexion.
Leung et al., 2012 [23]	Limber: accelerometer, IMU, strain gauge	Accelerometers: shoulders + IMUs: spine and neck, contained in hoodie; (stretch sensors on wrist)	Game-like positive and negative feedback regarding posture on computer	Proposal of two prototypes to encourage maintenance of good posture whilst sitting over the duration of the workday (*n* = 4)	Enable a minimally disruptive and highly engaging method for monitoring and correcting poor posture in an office-style workplace	Outcome limitation: Concerns with comfort, aesthetics and incorporation with work protocol; further testing required for validation No formal data provided.
Hermanis et al., 2015 [46]	9 axis IMU: accelerometer, gyroscope, magnetometer	Sensors contained within a 7 × 9 grid that is attached to the back of a vest	Real-time visual feedback via Android app	Proposal of Wearable Sensor Grid consisting of IMUs to monitor posture	No validation testing conducted	Outcome limitation: With no validation published as of yet, this remains a prototype with unknown validity
Giansanti et al., 2009 [3]	IMU: 3 uniaxial accelerometers, 3 gyroscopes	Sensor mounted at L5 (close to centre lf mass)	Real-time auditory feedback; sound volume correlating with degree of flexion	Proposal of using wearables and auditory feedback to improve postural control (*n* = 9)	Reported improvement in balance and decrease in energy expenditure with use of this auditory biofeedback wearable system	Comparability limitation: Specific auditory feedback requires intact hearing in users, this may limit use of this device in the elderly and those with hearing deficits; less related to spinal posture and more to postural control No data on sensor accuracy,
Millington, 2016 [22]	Lumo Lift: IMU sensor: tri-axial accelerometer, gyroscope, magnetometer Lumo Back: accelerometer Prana: sensor measuring posture and breathing	Lumo Lift: worn under clothes under the clavicle Lumo Back: waist Prana: waist	Lumo Lift: real-time vibrotactile feedback Lumo Back: real-time monitoring through smartphone app Prana: push alert reminders to sit/breath better and real-time monitoring through app	Qualitatively assess commercial wearables available for postural analysis	Haptic surveillance of posture enables shared responsibility of postural monitoring	Outcome limitation: Qualitative analysis of these devices, therefore no validation on the accuracy and validity of these devices in various clinical contexts
Felisberto et al., 2014 [13]	BodyMonitor: IMU: tri-axial accelerometer, gyroscope, magnetometer	1: upper torso 2: hip 3: leg No formal error rate, however was capable of detecting 70% of “incorrect activity” definitions.	No real-time feedback	Proposal of monitoring posture in the elderly with aim of decreasing premature nursing home admissions (*n* = 5, across multiple movement and orientation states)	Verification of using the wearable system for the identification of various body postures	Outcome limitation: Further testing required for validation; only tested identification of poor/good posture whilst sitting
Lin et al., 2016 [47]	Microelectro-mechanial tri-axial accelerometer	1: lower cervical spine 2: middle of the chest 3: L3 (centre of mass) 4: right waist 5: left waist Error rate in previously published work from group of 0.466 degrees.	Real-time visual feedback via smartphone app	Proposal and validation of wearable system incorporating five sensors affixed to a vest for real-time posture monitoring	Wearable system is comfortable, washable and easy to wear; all proposed functions of the system were validated	Selection bias: Tested in elderly subjects with the smartphone app driving technology anxiety. Total subjects not provided.
Voinea et al., 2016 [48]	IMU	Five sensors affixed to shirt in midline running from upper thoracic to lower lumbar spine	No real-time feedback	Proposal of model that converts orientation angles from the wearable system to calculate the curvature of the spine	Maximum error percentage < 5%, proposed mathematical model validated for reproduction of spine curvature; suitable for postural monitoring	Comparability limitation: Only uses one axis from the IMU; development to analyse all axes should further validate this system in kyphosis, lordosis and scoliosis. Total subjects not provided.
Kang et al., 2017 [35]	Smart garment: IMU sensors, metal composite embroidery yarn	IMU sensors: left and right shoulder, left and right waist. Anterior/posterior direction tilt angle error of less than 4 degrees.	No real-time feedback	Proposal of garment to measure postures; compared with motion capture camera system	Reported reasonable estimate of pitch and roll motion; feasible for postural monitoring	Comparability and outcome limitation; Posture estimates require an algorithm to compensate for the coupling of body motion
Charry et al. 2011 [49]	DorsaVi’s ViMove: IMU sensors (one tri-axial accelerometer, one single axis gyroscope)	1: L1 2: S1 RMS error range 1.9–2.5 degrees across flexion and lateral flexion and 4.1–5.2 degrees for twisting motion.	No real-time feedback	Proposal and assessment of accuracy of ViMove in measuring 3D orientation of lumbar spine (*n* = 2)	Once the raw inertial signals were processed by the Positional Algorithm there was a “good agreement” with Optotrak System	Selection bias: Only tested on two subjects; further research with a larger sample size required to determine if suitable for clinical use

There were over 30 devices identified, with nineteen studies detailing real time feedback systems (3, 6, 13, 18–22, 24–26, 29, 31, 34, 36, 38–40, 42). Among the included studies, ten studies had explicit aims of technological validation while most remaining studies others were limited as a proposal. Only seven studies employed the use of one sensor or single-platform with multiple sensors attached (3, 13, 20, 21, 35, 36, 40), with the vast majority requiring multiple sensors worn at a single time. Parameters measures ranged from aspects of balance, spinal positioning, motion analyses and incidence of pre-defined postural positions. Two studies were explicitly focused on the analysis of spinal motion during chair-sitting exclusively (6, 38). One study had an aim to identify the optimal sensor positioning using a spine-based system as opposed to clinical validation (16). No studies included any meaningful cost analyses. When provided, the accuracy rates of devices were high with error ranges within 5 degrees in the majority of capturing moments (> 85% of the time). Overall accuracy reporting is detailed later in the discussion.

Tables 2 and 3 detail the instruments used across studies within the wearable inertial units and the clinical populations in which testing was performed across the studies respectively.

**Table 2:** Summary of IMUs

Components	
Accelerometer	Measure proper acceleration
	- I.e. gravitational force (static) and sensor movement (dynamic)
	- At least one 1D accelerometer
Gyroscope	Measure angular velocity
	- At least one 1D gyroscope
Magnetometer	Measure all magnetic fields
	- Optional
Degrees of Freedom (DoF)	
9 DoF	3D accelerometer, 3D gyroscope & 3D magnetometer:
	- Most accurate type of IMU as able to measure proper acceleration, angular velocity and magnetic fields in three axes
	- Used in: PostureMonitor [34]; Zishi [39]; Xu’s wearable system [33]; Hermanis’ Wearable Sensor Grid [23]; LumoLift [46]; BodyMonitor [22]
6 DoF	3D accelerometer & 3D gyroscope:
	- Less accurate than 9 DoF IMUs as no magnetometer, therefore lower accuracy in determining sensor orientation
	- Used in: Giansanti’s wearable system [3]; SPoMo [6]; Wong’s wearable system [12]; Miyajima’s wearable system [20]
5 DoF	3D accelerometer & 2D gyroscope:
	- Less accurate than 6 DoF IMUs as gyroscope cannot measure in the third dimension
	- Used in Lou’s Smart Garment [44]
4 DoF	3D accelerometer & 1D gyroscope:
	- Less accurate than 5 DoF IMUs as gyroscope can only measure in one dimension
	- Used in DorsaVi’s ViMove [35]

**Table 3:** Summary of posture wearable applicability

Application	
Post-operative rehabilitation [25]	Falls [3, 13, 22]
- Reduced face-to-face hours with rehabilitation provider	- Detection of elderly falls within the home
- Tele-rehabilitation	- Improvement of postural stability to decrease the prevalence of falls
Treatment of MSK disorders	Workplace use
- Adolescent idiopathic scoliosis [40]	- Office workers [6, 16]
- Postural kyphosis [44]	- Construction workers [11.13]
Diagnosis	Comparison of treatments
- Pathological spine postures [24]	- E.g. spine operation types
- Assist in the clinical diagnosis and rehabilitation of other MSK disorders [9]	- Through pre-operative and post-operative monitoring [9]
Monitoring of disease progression	
- Parkinson’s Disease [43]	

Due to limitations in cohort differences and these recordable device parameters between studies it was not possible to meta-analyse data as a means of evaluating any expected postural based measures.

## Discussion

### Wearable technology

In 1994, Tanaka et al. proposed the first wearable system able to measure spinal posture without an observer. The system comprised of three electro-magnetic inclinometers measuring angle of inclination in the sagittal field. The inclinometers were strapped to the chest, thigh and calf. An analog-to-digital converter was incorporated to transpose measured angles into 4-bit digital signals allowing digital reconstruction of the trunk, thigh and calf inclination angles. The proposed system had an angular resolution of 12 degrees, suitable for categorisation of posture as “poor” or “good”. While Tanaka et al. reported “good linearity observed over the wide range of measured angle” there was no formal assessment of the accuracy of the system [12].

Since the proposal of such devices various prototypes capable of measuring spinal posture have been proposed. A wide range of technologies underpin these systems with the most commonly used being Inertial Measurement Units (IMUs). IMUs are generally comprised one or more accelerometers and one or more gyroscopes, and may also include one or more magnetometers (Table 2). Forces measured by accelerometers may be static, e.g. gravitational force, or dynamic, i.e. caused by moving or vibrating the accelerometer. The use of gyroscope permits precise measurement of angular velocity that is not influence by outer external forces detected via accelerometry. The absolute nature of gyroscopes, however, raises an issue of bias error due to drift. Unlike accelerometers, which use the gravity vector as a reference, gyroscopes do not have a reference and therefore are unable to reset to an initial state, thus leading to an accumulation of errors. This may be reduced through magnetometer integration as these calibrate IMUs with reference to the Earth’s magnetic field. However, as magnetometers cannot discriminate between the Earth’s and other magnetic fields they may be prone to interference from hard iron distortions [13]. As a result, IMUs general contain a combination of all three monitors in all three vehicle axis planes (i.e. Euler Angles): pitch (x-axis); roll (y-axis); and yaw (z-axis) [14].

Some wearables couple IMUs with other technologies to improve overall accuracy of derived data. Plamondon et al. [15], proposed a wearable that combined two IMUs linked by a potentiometer. The potentiometer was shown to greatly improve the validity of the system by assessing the relative longitudinal rotation between the two IMU. The same study reported that the inclusion of the potentiometer reduced all root mean square error to < 5 degrees, vastly improving data reliability [15]. This magnitude of error (< 5 degrees) appears to be the accepted standard throughout the literature. Our findings (Table 1) reveal a very high accuracy rate across different systems with error rates < 2 degrees and high agreement to control methods, usually in the form of video footage or fluoroscopy. It may be speculated that these high accuracy findings are affected by publication bias against less accurate devices.

Other wearables use only accelerometers without the addition of gyroscopes or magnetometers. Harms et al. [16] proposed a system utilising five accelerometers affixed to a shirt in various locations. Three sensors were placed along the spinous processes of C7, T10 and L5, thus allowing for the detailed assessment of forward-flexion of the entire spine. A fourth sensor was placed at the scapula and the fifth was placed at a more superior aspect of the shoulder, thus allowing for measurement of lateral flexion [16]. However, the system proposed by Harms et al. [16] employed a loose fitting shirt and increasing movement of sensors along the skin yielding large measurement errors in the T10 and L5-located sensors. In particular, measurement errors increased with increased angle of inclination. Overall, their study suggested > 84% to represent an acceptable magnitude of sensor orientation error (i.e. percentage of samples at a specific angle of inclination within 5 degrees of true inclination). The proposed system is therefore only suitable for measuring posture between 0 and 20 degrees for forwards flexion. [16]. Other technologies used in posture monitoring wearables include: strain gauges; flex sensors; fibre-optic goniometers; inductive sensors; ergonomic dosimeters [17–21]. These devices are briefly explored in Table 1, however at this stage are not validated for routine clinical use.

### Clinical applicability

Wearables measuring spinal posture have many possible clinical applications in the prevention, monitoring and treatment of chronic disease (Table 3). Mass production of smartphones, tablets and laptops have led to their incorporation into everyday life [22]. In office workers, long hours spent sitting in front of computers inevitably leads to poor posture. Ergonomic strategies to maintain correct posture, e.g. standing desks, have somewhat helped to reduce occupational risks to posture, however the unconscious deviation from intended correct posture is inevitable [23]. Wearable systems with the ability to monitor posture and provide real-time feedback alerting of sustained poor posture enable workers to correct their posture and hence decrease total time in poor postural states [9]. Abyarjoo et al. [14] have proposed an IMU-based wearable system for office workers that was reported to be sensitive enough to warn the user of poor postural states. This study was only a proposal and preliminary verification of the prototype, with studies assessing its validation in the office worker setting ongoing. This occupational environment represents a key health domain where such sensors may yield reduction in degenerative and traumatic postural changes on the basis of wearable derived preventative strategies. Such workers, particularly in the construction industry, are regularly exposed to repetitive strained postures and heavy lifting, establishing a clear focus population for future research. Yan et al. (2017) have proposed a wearable system with accompanying smartphone application producing auditory alerts upon detection of poor posture in the head, neck or trunk for longer than the acceptable holding time [24]. Like the system proposed by Abyarjoo et al. [14], this tool is also pending clinical validation.

Wearables measuring posture have a great potential for use in physical rehabilitation. Low back pain and other musculoskeletal disorders often require significant long-term rehabilitation to strengthen muscles and counteract postural deviations. Monitoring of posture using wearables may help to assist physical therapists ensure patients are properly executing rehabilitation exercises [25]. Furthermore, wearables may allow for reduced supervision by health professionals and the introduction of tele-rehabilitation via video conferencing. By using a wearable system at home, a physical therapist may be able to instruct and remotely monitor a patient’s posture in real-time. Tele-rehabilitation is likely to have the biggest use in those geographically far from medical services and those who struggle to leave their residence (e.g. the elderly) [26]. A systematic review of tele-rehabilitation by Kairy et al. [27] reported that patients and therapists perceived tele-rehabilitation as useful and convenient. It was also reported that tele-rehabilitation was less costly for the healthcare system, however a cost analysis was not provided.

Postural wearables have the potential to become the primary treatment modality for some musculoskeletal disorders. Bazzarelli et al. [28] proposed a wearable system for treatment of adolescent idiopathic scoliosis (AIS). Traditionally, AIS is treated using braces that exert passive force on the spine causing the wearer to actively pull away from induced pressure points. It is reported that the active muscle contraction induced is the most important therapeutic component. The system proposed enforced this active contraction through vibrotactile feedback that was shown to be effective in encouraging postural correction in those with AIS and claim a device error rate < 1% for measurements in the angular range of 0 and 70 degrees [28]. Lou et al. [29] have proposed a similar device utilising IMU sensors affixed to a garment for the treatment of postural kyphosis in adolescents. Like AIS, postural kyphosis is primarily managed with aggressive bracing aiming to strengthen back muscles and increase spinal flexibility. Their findings highlighted an excellent correlation (pearson co-efficient > 0.999; *p* < 0.05) in the comparison of sensor data to a standardised rotating wheel apparatus [29]. However although both systems have demonstrated adequate accuracy, data assessing long-term outcomes and longitudinal clinical use remains lacking. There are no current studies demonstrating the impact of such devices on correction of posture.

Other clinical applications for postural monitoring wearables include: detection of falls within the home; prevention of falls through improved postural control; assessment of Parkinson’s Disease severity [13, 16, 30]. The objective nature of wearables allows for their use in the aforementioned applications without the concern for subjective bias [31, 32]. With significant validation of postural wearable systems it may be possible for their use in the diagnosis of pathological spinal posture and the comparison of treatment modalities through the assessment of pre- and post-treatment postures [33].

### Practicality

While many of the proposed wearables promise the potential use in a wide variety of clinical applications, the biggest challenge remains in the lack of validation of these technologies. Most of the reviewed articles either solely proposed prototype designs or conducted preliminary verification of devices using very small samples over a short-term of time. In order to validate their use for long-term postural monitoring and improvement, larger and longer duration validation studies are required.

Compared to their physical activity monitoring counter parts, wearables measuring posture are significantly lagging behind in terms of commercialisation. While the aforementioned lack of validation studies is a major cause, the need for more than one sensor in determining spinal posture presents a significant challenge regarding practicality. Although some of the wearable systems proposed only used one sensor, Fathi et al. [34] reported an optimum number of three IMU sensors for accurate classification of posture. Their findings indicated that the addition of more than three sensors did not achieve a statistically significant improvement in the accuracy of postural analysis data [34]. Multiple sensors require connection via wires and attachment to the body, often by strapping they may be challenging for the user to remove and reattach [26]. The key to the commercialisation of these devices is finding the perfect middle ground between accuracy and wearability. Hence, it is understood that this is the rationale for the popular choice of three sensors as seen in many of the proposed devices.

One method to make multiple-sensor wearables more practical is the incorporation of sensors into smart textiles. Kang et al. [35], proposed a wearable garment that contained four IMUs incorporated within stretchable conductive yarn. The conductive yarn functioned to transmit signals from the sensors to the processor and also allow for battery power transmission. The wearable garment was reported to provide a reliable estimation of postural tilt of the torso over 1 h. There was strong linearity demonstrated in pitch and roll directions, with both producing an R^2^ > 0.973. There were, however, increased errors in measurements over 1 h. It is suspected that this increase in estimation errors occurred due to the relative motion of IMU modules against the body [35]. Despite the stretchable nature of the fabric, future improvements to this system must address this issue of sensor movement.

Millington [22] reviews the three commercially available wearables able to monitor spinal posture: Lumo Back; Lumo Lift; Prana. The Lumo Back is an accelerometer-based sensor measuring 3.9 in. and is worn at the waist. It monitors lower back posture and encourages self-surveillance via a smartphone application but does not provide real-time warnings of sustained poor posture. The Lumo Lift is a smaller and more discreet device, measuring 1.74 in. and clasped under clothes just inferior to the clavicle. It measures upper back posture allows users to switch between posture alert mode, where vibrotactile feedback is provided in response to sustained poor posture, and coaching mode, where a vibration is delivered as soon as poor posture is assumed. The Lumo Lift also monitors step count, distance and energy expenditure. The Prana is a disc-shaped sensor measuring 1.25 in. that is worn at the waist. It measures breathing and posture and sends notifications reminding users to maintain neutral posture [22]. Millington’s review of these three devices is only a qualitative analysis and lacks reference to significant quantitative validation. A comprehensive review of the literature also failed to find published validation trials for these devices. Hence, further research assessing their impact long-term is required.

As devices continue to improve in accuracy and achieve validation we believe the next step towards integration of such wearables would be to establish a link between data output and diagnostic predictability while optimising costs of integration. The implication a diagnostic algorithm in this regard would facilitate healthcare delivery without the need for regular consultation and the potential to remotely highlight populations requiring urgent intervention. This remains a key focus for our own research institution at this stage.

## Conclusion

This is the only study to specifically review wearable devices that monitor spinal posture. This review reveals that spinal posture can be measured through the use of various technologies but that there is limited data regarding the validation of the same. More research into the accuracy and long-term outcomes of these devices is required for a greater understanding of their clinical applicability. Furthermore, improvements regarding practicality are required before commercialisation and mass uptake can be considered.

## Abbreviations

AIS: Adolescent idiopathic scoliosis
DoF: Degrees of Freedom
IMUs: Inertial Measurement Units
PRISMA: Preferred Reporting Items for Systematic Reviews and Meta-Analyses Guidelines
